# New insights into the pathology of pulmonary hypertension: implication of the miR-210/ISCU1/2/Fe-S axis

**DOI:** 10.15252/emmm.201505160

**Published:** 2015-04-07

**Authors:** Haiyang Tang, Ramon J Ayon, Jason X-J Yuan

**Affiliations:** Division of Translational and Regenerative Medicine, Department of Medicine and Department of Physiology, The University of Arizona College of MedicineTucson, AZ, USA

## Abstract

Elevated pulmonary arterial pressure in patients with pulmonary hypertension (PH) is mainly caused by increased pulmonary vascular resistance (PVR), due primarily to sustained pulmonary vasoconstriction and excessive pulmonary vascular remodeling. According to the current classification, PH has been classified into five categories based on etiology (Simonneau *et al*, 2013). Among them, group 1 or pulmonary arterial hypertension (PAH) is a rare but progressive and deadly disease affecting ∽1–10 per 1 million people. Despite expanding treatment options to ameliorate patients' symptoms, PAH remains a devastating disease with a poor long-term prognosis.

See also: K White et al (June 2015)

The exact pathogenic mechanisms of PAH are complex and poorly understood, yet a number of abnormally expressed genes and regulatory pathways contribute to sustained vasoconstriction and vascular remodeling of the distal pulmonary arteries.

miRNAs have attracted tremendous interest as important epigenetic regulators of physiological and pathological events in the cardiovascular system. The aberrant expression of miRNAs has been detected in animal models and patients with PAH (Caruso *et al*, 2010; Courboulin *et al*, 2011). Down-regulated expression of miRNAs including miR-22, miR-30, and let-7f was found in lung tissues from two rat models: monocrotaline (MCT)-mediated PH and chronic hypoxia-induced PH, while the expression of miR-322 and miR-451 was up-regulated (Caruso *et al*, 2010). Another group reported that the increase in STAT3 activation in the MCT-injected rat model decreased the expression of miR-204, which thereby up-regulated the activation of STAT3 and NFAT, thus allowing PASMC proliferation and resistance to apoptosis (Courboulin *et al*, 2011). Kim *et al* reported that the decreased expression of apelin (APLN) in PAH pulmonary arterial endothelial cells (PAECs) down-regulates miR-424/503, which directly suppresses FGF2 and FGFR1 and decreases the phosphorylation of ERK1/2 (Kim *et al*, 2013). A recent study by Chen *et al* reported that suppression of miR-17-92 in PASMCs attenuates experimental PH and indicated that miR-17-92 inhibits PDLIM5 to induce the TGF-β3/smad3 pathway, contributing to SMC differentiation and the pathogenesis of PAH (Chen *et al*, 2015). These studies suggest that differential expression of miRNAs contributes to alteration of genes in the development and progression of PH (Fig1). Continued efforts in elucidating the role of miRNAs in regulating target genes that are involved in the development and progression of PH are important to fully understand the molecular mechanisms leading to this disease.

**Figure 1:**
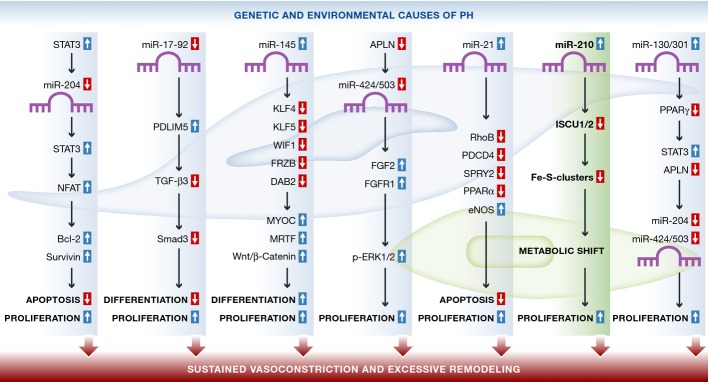
MicroRNA regulatory pathways implicated in pathogenic mechanisms of pulmonary arterial hypertension (PAH) Dysregulation of miRNAs during the development of pulmonary hypertension (PH) alters the expression of downstream targets leading to changes of pulmonary arterial smooth muscle (PASMC) and/or endothelial (PAEC) cell proliferation, differentiation, apoptosis, and migration, which contribute to the sustained vasoconstriction (due to PASMC contraction) and excessive vascular remodeling (due mainly to PASMC/PAEC proliferation and migration) and eventually lead to elevated pulmonary vascular resistance and pulmonary arterial pressure.

In this issue of *EMBO Molecular Medicine*, White and colleagues provide evidence that alterations of the miR-210–ISCU1/2 regulatory axis can cause Fe-S deficiencies *in vivo* and contribute to the development of PAH (White *et al*, 2015). The authors found that the expression of miR-210 was up-regulated in lungs from several distinct PH models including a genetic one (von Hippel-Lindau knockout mouse), a chronic hypoxia-induced PH model, the hypoxia + Sugen model, and inflammatory PH models (IL-6 transgenic mouse and chronic *S. mansoni*-infected mouse). However, the direct targets of miR-210, ISCU1/2 were down-regulated in pulmonary vasculature in PH mice and patients with PH. In agreement with their previous report, the authors confirm that Fe-S integrity in pulmonary arterial endothelial cells is directly mediated by the miR210/ISCU1/2 axis and the decrease in Fe-S levels is due to activation of miR-210 (Chan *et al*, 2009). Down-regulation of ISCU1/2 was also found in hypoxic cells and lung tissues from PH mice and patients with PAH. Several lines of evidence in this study suggest that chronic induction of miR-210 during the development of PH suppresses ISCU1/2 as a primary target, leading to disruption of Fe-S biogenesis, mitochondrial metabolism, and pathologic alteration of the proliferative and oxidative states of the pulmonary vasculature. Accordingly, miR-210 knockout or antisense inhibition of miR-210 significantly attenuated hypoxia + SU5416-induced PH and pulmonary vascular remodeling. Injection of miR-210 mimics or siRNA knockdown of ISCU1/2, which consequently decrease Fe-S integrity without a reduction of total mitochondrial DNA content, resulted in elevated right ventricle systolic pressure and promoted pulmonary vascular remodeling. Collectively, the data provide a molecular mechanistic link between the miR-210/ISCU1/2/Fe-S axis and PH. In addition, the finding that manipulation of miRNA expression affects experimental PH offers great promise for miRNA-based therapeutic strategies in human PAH.

This study by White *et al* also defines a novel signaling cascade that may be a crucial pathogenic lynchpin of pulmonary vascular disease and also advances our understanding of mitochondrial metabolic dysfunction in PH. In the pulmonary vasculature, mitochondria function as oxygen sensors and stabilize hypoxia-inducible factors (HIFs) by generation of reactive oxygen species (ROS) at mitochondrial complex III under hypoxia, resulting in pulmonary vascular remodeling and pulmonary hypertension (Chandel *et al*, 2000). White *et al* (2015) found that Fe-S integrity, but not total pulmonary iron content, was significantly down-regulated in PH mice and lungs from PH patients. These findings, as the authors suggest, offer an alternative paradigm of pathogenesis whereby mitochondrial respiratory activity is dysregulated, independent of respiratory complex protein expression but critically dependent on miR-210, ISCU1/2, and Fe-S integrity.

In concert with previous findings on the miRNAs and PH, this study will undoubtedly open up new pipelines of research and novel therapeutic approaches for this disease. However, these exciting studies raise several important issues. First of all, what are the underlying mechanisms responsible for regulation of the expression and activity of miRNAs themselves? Most studies have focused on the aberrant expression of miRNAs and downstream target genes during disease. However, little is known about the molecular and cellular mechanisms that underlie the dysregulation of miRNA in the development of PH. Hypoxia and HIF, major contributors to the pathogenesis of PH, have been thought to act as crucial triggers for miRNA dysregulation in PH. Many efforts have been made aiming to identify hypoxia-regulated or HIF-regulated miRNAs. miR-210 is consistently up-regulated in hypoxia conditions as indicated in all the separate studies (Ivan & Huang, 2014). Therefore, it would be interesting to determine how the miR-210/ISCU1/2/Fe-S axis is regulated by hypoxia and HIF and its implication in the pathogenesis of PAH. Another issue is that these profiles, based on microarray analysis and quantitative PCR, vary substantially and with limited overlap between studies. Although such inconsistences can be explained by differences in cell types, animal models, disease stages, techniques, and conditions in which they were performed, further identification and validation of miRNAs are needed to clarify their roles in the pulmonary vasculature. Additionally, whether there is a connection or interaction among these well-defined miRNAs implicated in PH remains unclear. One example showed by the same group is miR-130/301, which suppresses miR-424/503 in endothelial cells and suppresses miR-204 in smooth muscle cells during disease (Bertero *et al*, 2014). Further studies are also needed to provide a better understanding of the complex and interactive miRNA modulation network in the development of PH. In summary, this comprehensive study not only provides a novel molecular mechanistic link between the miR-210/ISCU1/2/Fe-S axis and PH, but also carries broad translational implications for defining the metabolic origins of PH.
